# Studies of Expertise and Experience

**DOI:** 10.1007/s11245-016-9412-1

**Published:** 2016-07-15

**Authors:** Harry Collins

**Affiliations:** Cardiff University, Cardiff, UK

**Keywords:** Expertise, Studies of expertise and experience (SEE), Expertise space diagram, Three dimensions of expertise, Interactional expertise, Imitation Games

## Abstract

I describe the program of analysis of expertise known as ‘Studies of Expertise and Experience’, or ‘SEE’ and contrast it with certain philosophical approaches. SEE differs from many approaches to expertise in that it takes the degree of ‘esotericity’ of the expertise to be one of its characteristics: esotericity is not a defining characteristic of expertise. Thus, native language speaking is taken to be an expertise along with gravitational wave physics. Expertise is taken to be acquired by socialisation within expert communities. Various methods of analysis are described.

## Preamble

The Muenster meeting was intended to be interdisciplinary hence this preamble. Is what I do here philosophy or sociology? My preferred answer is that the question is misconceived. I am not a trained philosopher but those doing sociology degrees in the 1960 and 1970s in the UK had quite a bit of philosophy in their course—ethics, philosophy of science and philosophy of social science. So I got a smattering and it was the part of the subject that, along with sociology of knowledge, I found most interesting. By the time I finished my degree I was ‘a Popperian’ but then events caused me to read Peter Winch’s little book, published in 1958, *The Idea of a Social Science.* Winch was a philosopher and his book was about the implications of Wittgenstein’s later philosophy for sociology.^1^ Its well known conclusion was that the deep questions of sociology were really ‘misbegotten epistemology’. In 1968 I struggled with the book but after many readings I began to get the idea, went back to the original Wittgenstein and found I could understand it quite easily from a Winchian perspective, and my life changed. Nearly everything I have done since has been inspired by what I learned then, so philosophy is the foundation of pretty well all my work.

What Winch argues, totally convincingly as I see it, is that peoples’ actions and peoples’ concepts are two aspects of the same thing. You cannot understand the actions without the concepts and you cannot understand the concepts without the actions. Around page 120 Winch explains this in terms of the actions of surgeons in an operating theatre and their relationship to the idea of ‘germ’. *Inter alia* he invents the notion of scientific paradigm 4 years before Kuhn’s ‘*Structure*…’ which was published in 1962. What is less convincing is that this means sociology is misbegotten epistemology because one could equally conclude from the brilliant identification of actions and concepts that epistemology was misbegotten sociology (Winch on his head). The only reasonable conclusion is that when one is doing one, one is also doing the other (Winch on his side). That is why I think the question about whether this paper is sociology or philosophy is misconceived. Let me add that what I think about Wittgenstein is the same as what philosopher David Bloor thinks about Wittgenstein, that I have written a book with a philosopher (Martin Kusch) who also thinks the same way and, of the roughly 60 papers that I have published over the last 10 years, around 35 of them have been in philosophy outlets—see my entries in the bibliography to get a sense of these—so even I am not sure if I am more philosopher than sociologist.^2^

But let me also add that though I consider all my work has been inspired by philosophy and that much of it is as much philosophical as sociological, I probably would not do very well in a philosophy exam and some of what I do is still very different to many of the things philosophers do. Furthermore I use lots of tables and figures which philosophers don’t. Thus, I was disappointed, but not astonished, that after my presentation at the Muenster meeting the first remark addressed to me was: ‘that’s all very clever but what is it all for?’ The implication of this remark being, I think, that the only proper method for philosophy—the only method that is ‘for anything’—is armchair investigation of the necessary and sufficient conditions for something to be called ‘expertise’ or someone to be called ‘an expert’. I have nothing against armchair exploration since I do a lot of it and I believe that in virtue of our being fully socialised members of our society we already know a huge amount about those societies and their concepts and actions (and this was the basis of my hero Wittgenstein’s later work), but I think we have to be constantly vigilant in case armchair intuitions are misplaced. There is also *nothing wrong* with getting out of the armchair and doing experiments and what are, in regular academic parlance, referred to as ‘sociological’ investigations of one group of experts or another. I think, in other words, that we must be careful that our loyalty to our disciplines, and especially our loyalty to particular corners of our disciplines, does not divert us from using any tool that helps us learn about, in this case, expertise. Thus, it seems to me that a large group of philosophers’ intuitions point strongly to the idea that an expert is someone with more true and justified beliefs than non-experts while the intuitions of another, partially overlapping group of philosophers, direct them to believe that an expert is someone with hard-won esoteric skills or understandings. But I think both of these intuitions are wrong and that a bit of sociological-looking, thinking, and acting, along with new kinds of philosophical thought encouraged by looking, and acting, reveals them to be wrong; empirical examination of experts’ ways of being in the world can enliven and refresh even philosophical thought. Our responsibility is not just to analyse the intuitions that come to us in the process of socialisation into our societies but, sometimes, to change them.

## Domains of Expertise

What I am going to describe here comes under the label of ‘Studies of Expertise and Experience’ or SEE. SEE has been building for about 15 years.^3^ SEE starts with an approach to expertise which is based on Winchian/Wittgensteinian ideas. The approach takes it that there are ‘forms-of-life’ (cultures or paradigms) characterised by certain ways of going on and ways of thinking and that those who are fluent in these ways of going on and thinking are experts in those domains.

**Fig. 1:**
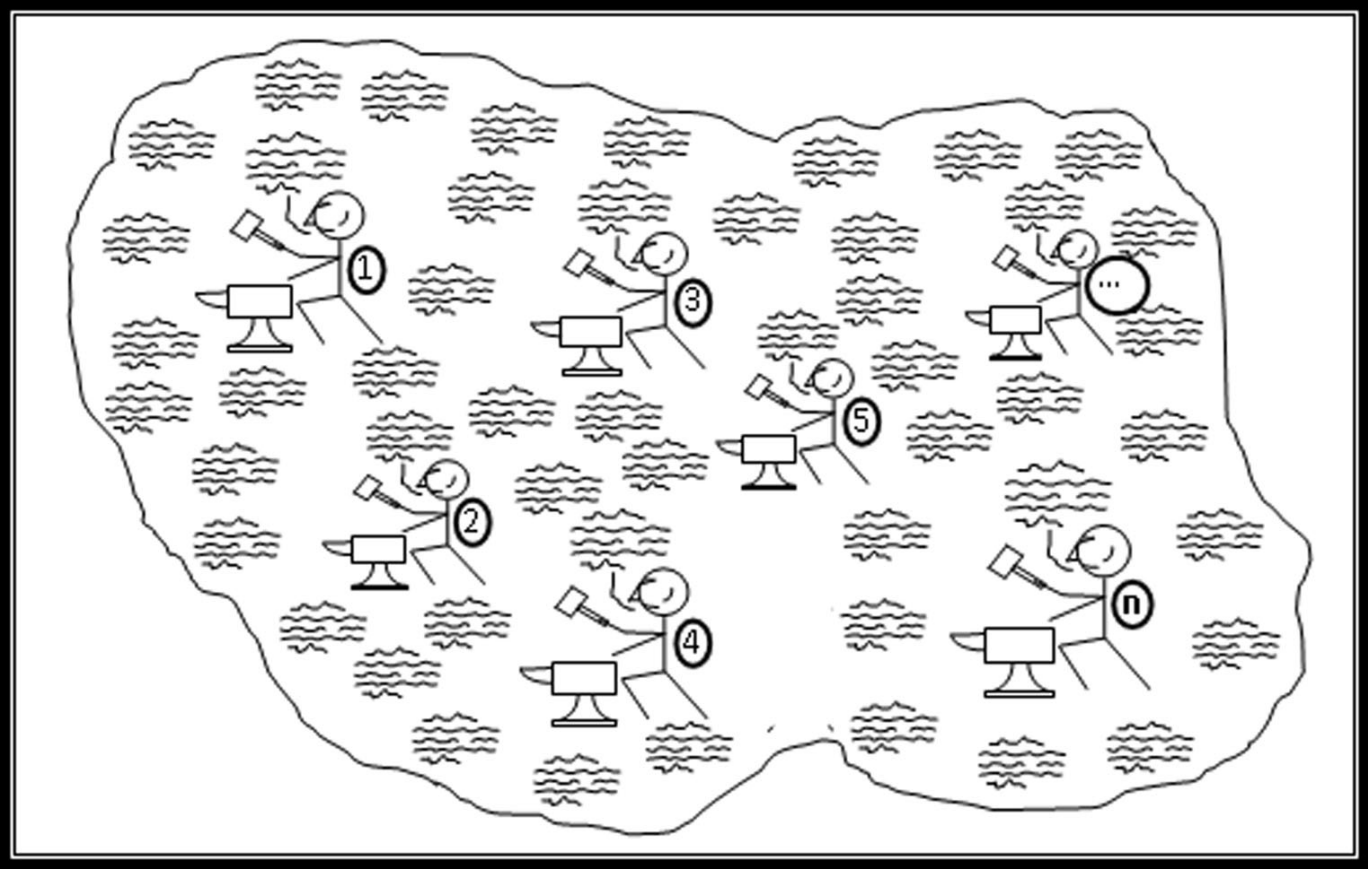
A form-of-life such as that of gravitational wave physicists

To become an expert in some domain is a matter of becoming embedded in the social life of the domain, acquiring what is to a large extent, tacit knowledge, so as to internalise the associated concepts and skilful actions to the point of fluency. Figure 1 is a sketch of a form-of-life—originally drawn to represent the author’s long-term embedding in the society of gravitational wave (GW) physicists—now a billion-dollar international project. It shows members engaged in spoken discourse (the packets of waves) and in actions such as building bits of gravitational wave detectors, doing calculations and publishing papers as represented by the hammers and anvils. Each one is a specialist at some particular practical activity as shown by the different numbers on the hammerers’ knapsacks, while their specialist actions are coordinated via a common set of concepts and a common spoken discourse—the ‘practice language’ of gravitational wave physics which is the waves.

Notice that a similar diagram could be drawn for any expertise. There is nothing here about whether the concepts thus absorbed lead to true beliefs nor about whether the forms-of-life, or domains of expertise, are esoteric or ubiquitous. I can be an expert in astrology just as much as I can be an expert in astronomy and I can be an expert in speaking English just as much as I can be an expert in linguistics. This means that these domains are found at many scales and that smaller ones are embedded in larger ones.

## The Fractal Model

**Fig. 2:**
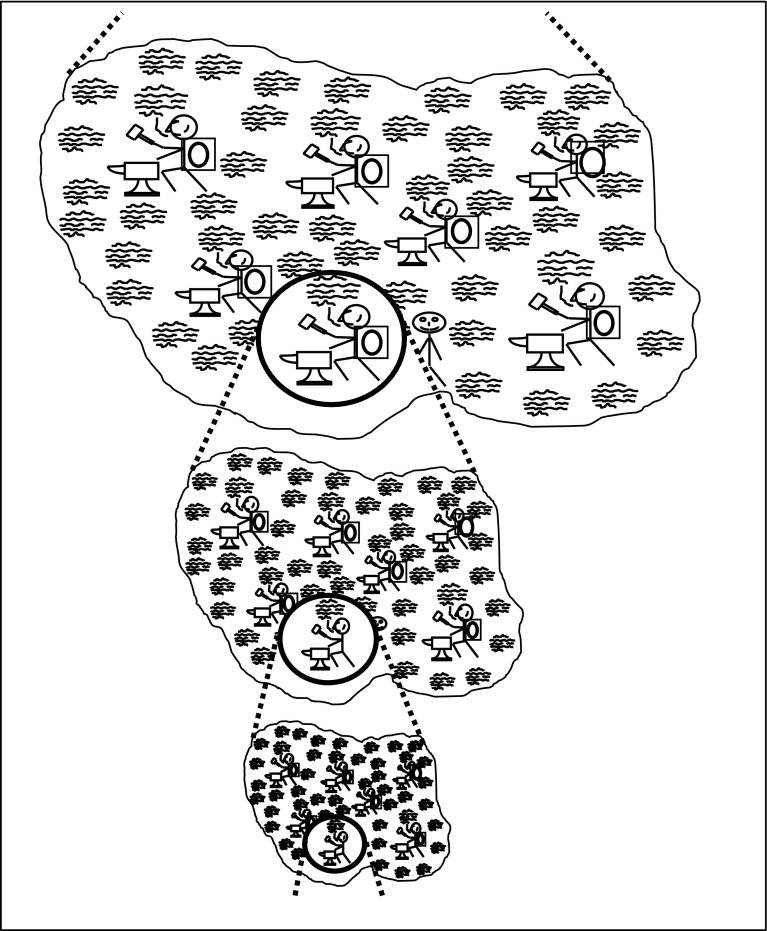
The fractal model

Figure 2 represents what we call the ‘fractal model’ of expertises. The term is meant to indicate that the same kind of basic structure and analysis applies to expertises at all levels. The top level of Fig. 2 might represent GW physics in which case the second level down might be the form-of-life of interferometer-builders and the third level the form-of-life of interferometer-mirror suspension designers. But there are levels above and the same diagram could represent English speakers at the top, scientists below and physicists below that, with GW physicists further down still below the frame. Furthermore, if we suppose the top level is physicists, then group 1 might be Christians and group 2 might be Hindus with special types of Christian and Hindu physicists below them and so on; the problem is that forms-of-life cross-cut each other.

**Fig. 3:**
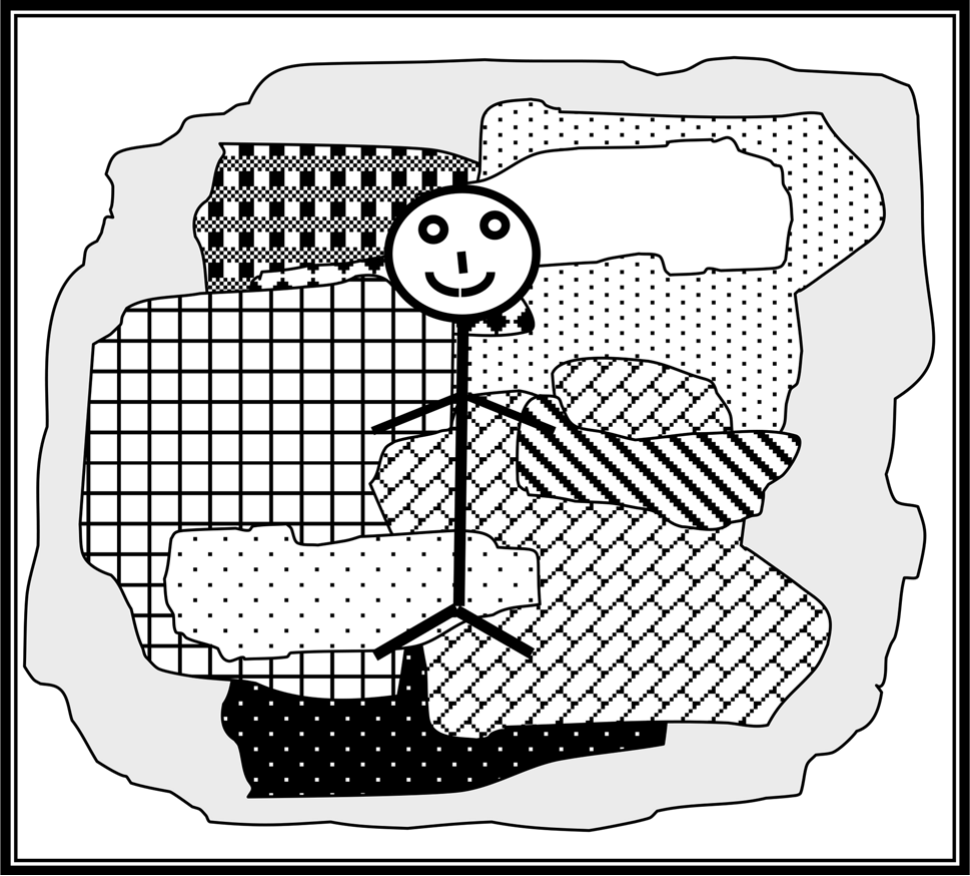
The individual as a collection of collectivities

The overall position adopted by SEE is that the way to understand us is to see us as a collection of forms-of-life: the atom is the collectivity, the individual is the molecule constructed from many collectivities as in Fig. 3.

Here the largest shape might represent, say, native English speakers with the smaller shapes representing GW physicists, cricketers, Christians and so on. Of course, there is a similar diagram for native French speakers so the whole thing is multi-dimensional but even if we cannot draw it, or even quite grasp its complexity, it gives us a way of thinking about individuals’ expertises and competences being a matter of embedding in forms-of-life even though the domains are intricately interwoven at many different scales and across many dimensions.

## The Three Dimensional Model of Expertise

As can be seen a crucial difference between SEE and many models of expertise is its indifference to scale and therefore ubiquity of expertise. In contrast to many philosophical and psychological models, SEE takes the ‘esotericity’ of an expertise to be a contingent matter rather than an essential property. Stage theories of expertise such as those of, Dreyfus and Dreyfus, or Chi, correctly describe some expertises but not others. This is because some expertises do not always pass through the standard stages (think of learning to balance on a bike or learning to articulate ‘tongue-twisters’) and because sometimes the stages are integral with growing out of infancy.^4^ The 10,000 hours of self-conscious practice model does not always work for similar reasons. Most fatal of all for the idea that expertise is necessarily esoteric is that the same expertise can be esoteric at one time or place and ubiquitous at another. Thus, fluent English speaking is not-counted as an expertise in England but is a valuable skill in France while car-driving and word-processing were esoteric skills when cars and desk-top computers were first developed but are now widespread. The greatest damage caused by the ‘essentially esoteric’ view of expertise has arisen from mistaking ubiquitous expertises, such as native fluency in a language, as not really expertises at all because everyone possesses them. Thus did at least one set of ludicrous misunderstandings of the power of computers come into being, a misunderstanding that is still present in science fiction representations of robots where fluent speech is never a problem even though in the real world of computing fluent speech is a distant dream.

**Fig. 4:**
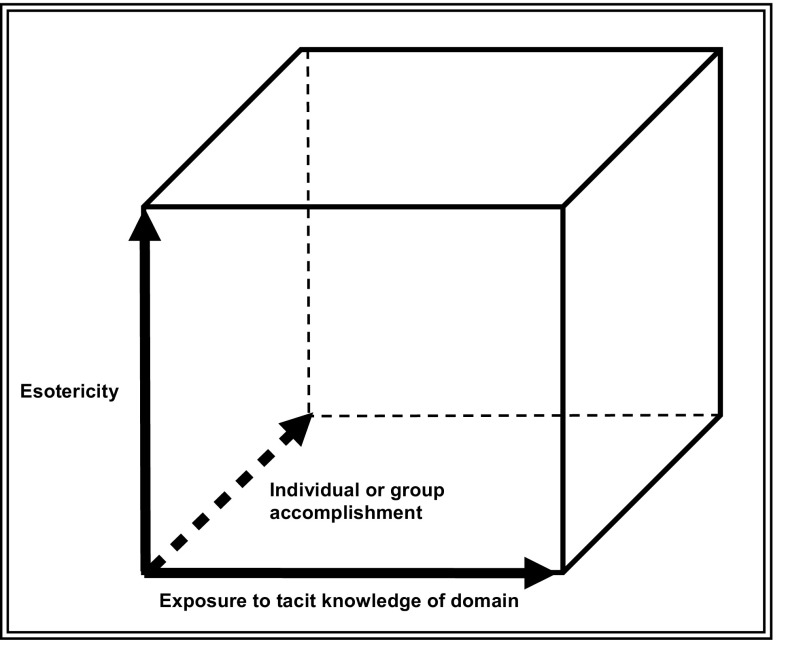
Three dimensional model of expertise

Understanding of expertise as essentially orthogonal to its ‘esotericity’ along with the SEE model of the acquisition of an expertise enables us to construct a three dimensional model of expertise—an ‘Expertise Space Diagram’—as shown in Fig. 4.

The front-to-back ‘Z-axis’ represents the usual philosophical or psychological ways of thinking about expertise—a matter of increasing individual accomplishment that may pass through certain definable stages. To this we can now add the, left-to-right, X-axis, which represents the extent to which an individual has access to the tacit knowledge of the domain in question such that he or she can gain fluency. Figure 1 shows one stick figure—the one toward the top left—who has no number indicating a practical specialism. We can think of this figure as a novice who is entering the field and has only started to acquire the language and the practical abilities and so has not yet developed into a specialist. If the novice is to succeed they must have good access to the tacit knowledge and will move to the right as they gain it. The, vertical, ‘Y-axis’ represents the esotericity of the domain, native English speaking being low down in English-speaking countries, GW physics being high in all known countries, though one can just about imagine a society where gravitational wave physics was taught from the cradle and in which it would be a ubiquitous expertise.

**Fig. 5:**
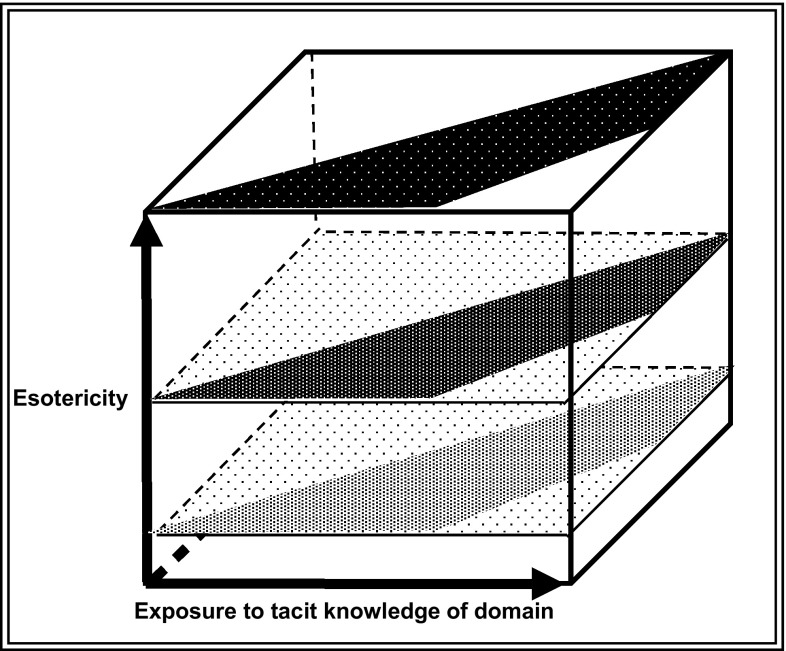
The expertise space diagram and car-driving

The expertise space diagram can be used in a number of ways. Figure 5 shows ‘surfaces’, in this case representing car driving. The top surface is represents racing-driving, an esoteric expertise. The back left hand void in these surfaces results from the impossibility of going far on the Z-axis without going far on the X-axis—i.e. acquiring tacit knowledge. There may be some skills where it is all, or nearly all, a matter of explication but they are hard to think of. The front right hand void is there because if a novice sticks around for a long time without starting to learn things—backward movement on the Z-axis—he or she is likely to be excluded from the company of experts. I guess the first of these voids is, roughly, philosophical and the second one is, roughly, sociological, but I must admit they both come from armchair consideration, though one based on lots of experience. The top surface could also represent car-driving when horseless carriages were first invented. The lowest surface represents ordinary driving in Western societies where the expertise is nearly ubiquitous. The middle surface can be an in-between position in time, in specialist driving skill (e.g. lorry-driving) or represent some location where there are few cars—some developing society.

**Fig. 6:**
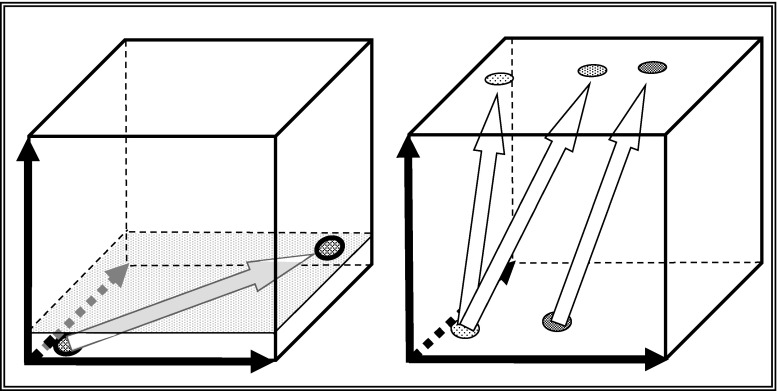
The expertise space diagram and educational trajectories

Figure 6 shows educational trajectories. The left of these two is kindergarten where infants are being taught to acquire a range of skills—language, proper social interaction etc.—that are ubiquitous in the society in question. The rightmost of the two diagrams represents university education—distance learning, normal face-to-face degrees with more acquisition of tacit knowledge and Ph.D., which involves quite a bit more tacit knowledge.

## The Periodic Table and Interactional Expertise

**Fig. 7:**
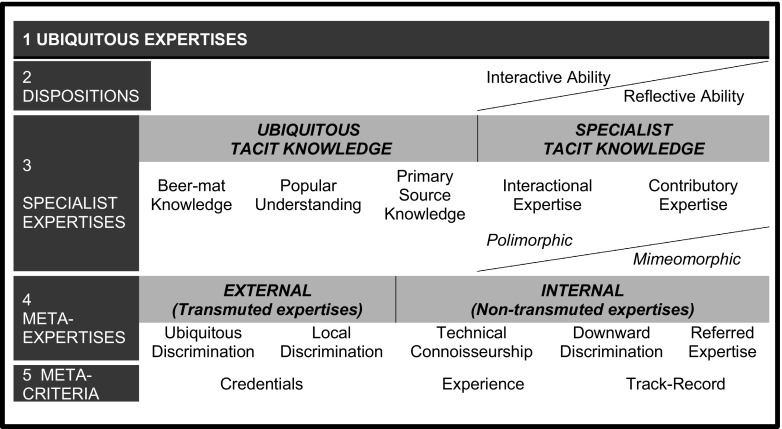
The periodic table of expertises

The Periodic Table of Expertises (Fig. 7) is an attempt to list, exhaustively, all the types of expertise there are that can bear upon questions of technological decision-making in the public domain, from ubiquitous to specialist expertise. We also include meta-criteria—the qualities of experts that members of the public might take into account in deciding whose view to trust but we consider these are not of great importance. More important is general social knowledge of in what sections of society to place trust in respect of technological matters: in general, if you want to know about stars, place trust in astronomers rather than astrologers, and so forth.^5^ This kind of understanding is found in the meta-expertise line: meta-expertise is expertise about experts. For example, ‘local discrimination’ is important for ‘whistleblowers’. Of particular importance is the division between Primary Source Knowledge and the rightmost entries in the specialist expertise row which refer to what are normally thought of as experts. Primary Source Knowledge is information obtained by those with only ubiquitous tacit knowledge (fluency in the language, general education up to and including university level and an understanding of libraries and other sources) who persevere with reading the professional journals. We show that the meaning of a published paper cannot be understood without understanding its location in the social milieu of the relevant technical domain. Thus there are published papers that are indistinguishable from all the others in a journal yet which the professionals in the domain simply ignore. The public have no chance of understanding this and that is one reason why obtaining knowledge from the internet without further back-up is unreliable. The table has been explained at length elsewhere (see “Bibliographical Appendix”) so here I concentrate on its most novel and contentious component, ‘interactional expertise’.

## Interactional Expertise

As can be seen, interactional expertise is one of two kinds of specialist expertise that depend on possession of the tacit knowledge of the specialist domain. The other, ‘contributory expertise’ is what we normally mean when we talk of experts—these are people who exercise their expertise by contributing to their specialist domain.

**Fig. 8:**
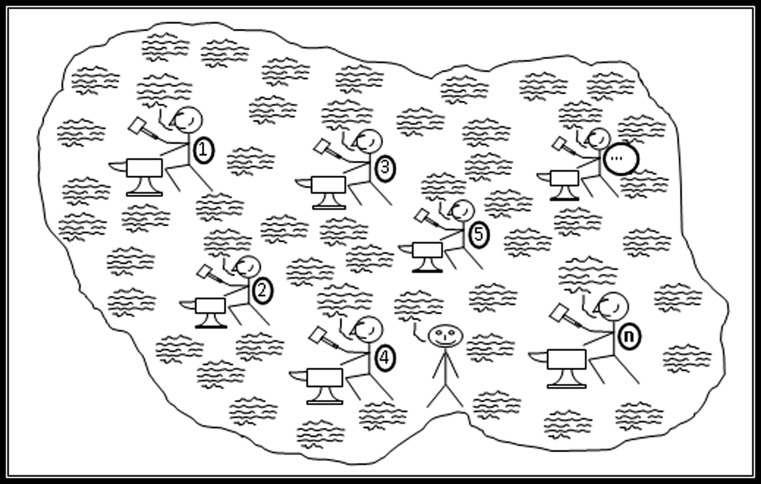
An interactional expert

Interactional expertise can be understood by referring back to Fig. 1 and the stick figure at the top left who we have already used to represent a novice entering the field and beginning to acquire both the spoken discourse (the wavy lines) and one or other practices (a hammer and anvil) pertaining to the domain. Figure 8 is the same as Fig. 1 except that here we introduce an additional stick figure, roughly bottom central, who has no hammer and anvil but acquires only the spoken discourse of the domain. Such a person is an interactional expert. The competences of such a person are represented by someone like Collins who has embedded himself in the domain of GW physics for more than 40 years with an especially intense 10 years of interaction from the mid-1990s during which he travelled to most GW conferences held around the world and continually emailed to friends and acquaintances in the field. By the late 1990s Collins found he could speak fluently and technically to GW physicists about GW physics even though he had never actually done any GW physics in the sense of building apparatus, doing calculations or otherwise contributing to the theory or helping to write papers for publication. He had become an interactional expert, or as we would now say, a ‘special interactional expert’ since we now believe that all contributory experts must be interactional experts in order to learn their craft.

This idea immediately raises a number of questions. What kind of expertise is this? How is it possible for someone to acquire expertise in a domain without fully engaging with it? There is now a considerable literature debating these points but here I will try to provide some simple answers.

This kind of expertise is more than ‘talking the talk’ while not being able to ‘walk the walk’ because it is a kind of expertise that enables one to make sound technical judgments that pertain to the domain: it is best thought of as ‘walking the talk’. A little more thought reveals that this is the main component of the expertise possessed by the managers of such large scale projects since they have make decisions about which technologies to pursue that will be worthy of the respect of the scientists and technologists they manage but, generally, without making day-to-day technical contributions to the field they are managing. A little more thought than that reveals that it is an expertise that is used in peer review where the reviewer will rarely have practised the practices whose worth is being judge. The same applies to committee meetings judging technical issues. And along with this is the *observation* that committee meetings that make technical decisions (for example, those which Collins observed taking place in GW physics including high-level meetings at the US National Science Foundation) do not involve calculations and experiments, they involve talk which, at best, reports the overall outcome of calculations and experiments—very much within the compass of the purely interactional expert (but one has to attend the meetings to see this happening). Still more thought reveals that it is the kind of expertise without which the division of labour between specialists in a domain like GW physics would be impossible because each specialist learns to execute only one or two narrow practical specialties but must coordinate their work in those specialties with that of all the other specialists and this is done by discourse: the wavy lines are common to everyone, the hammers and anvils are not. And this, in turn, explains how it is possible for an outsider who is not fully engaged with a specialist domain to acquire expertise in that domain; it is because the only difference between the outsider and the novice is that the outsider isn’t given one of the numbered hammers and anvils to work with. The novice, note, is given only one hammer and anvil, not all of them, so the very notion of there being a practice pertaining to the field is misplaced—there are many practices of which novices learn only one so the outsider is not much worse off than the novice. But do note that acquiring interactional expertise is a long and hard process. (Is this sociology or philosophy? It arises out of hanging around with GW physicists and almost certainly would not have arisen without this social experience beyond the armchair. On the other hand, the last paragraph is *mostly* a matter of thinking about things.)

The notion of interactional expertise is, of course, a departure from SEE’s Wittgensteinian/Winchian starting point as it separates what can be learned from spoken discourse alone from what can be learned from the mixture of language and practice that constitute a form-of-life—we call this the ‘separation principle’. Working from the precepts of SEE also leads to the conclusion that the notion of interactional expertise is fundamental to an understanding of the workings of society. For societies as a whole to work as they do, people must coordinate their actions just as they do in the specialist domain of GW physics. Since individuals cannot each practice each other’s practices, mutual understanding and coordination comes through interactional expertise making language, as though we did not already know it, central to human social life and quite different to animal life.^6^ It also makes the philosophically fashionable emphasis on practice as opposed to language wrong-headed—a conceit that seems to have arisen out of treating language as a formal set of propositions rather than a linguistic practice as heavily invested with tacit knowledge as any other practice.

## Imitation Games

**Fig. 9:**
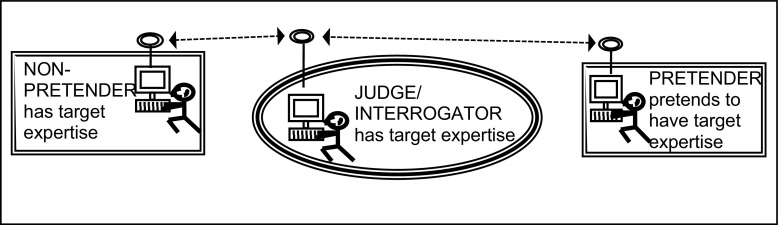
The basic imitation game

Now we move to something that is hard to call philosophy—experimental tests. What we have done is to adapt the famous Turing Test. In the Turing Test a hidden computer and a hidden person are questioned by ‘judge’ and if the judge can’t tell which is which we say the computer is ‘intelligent’. There is a lot of complexity underlying that innocent description but here let us simply note that Turing based the test on the imitation game, in which a concealed man pretended to a woman (or vice versa) while a judge asked question of him and a concealed woman and tried to tell the difference. We can say that if the man passed the test then it indicated the possession of interactional expertise in femaleness; femaleness was the target expertise (Fig. 9).

**Fig. 10:**
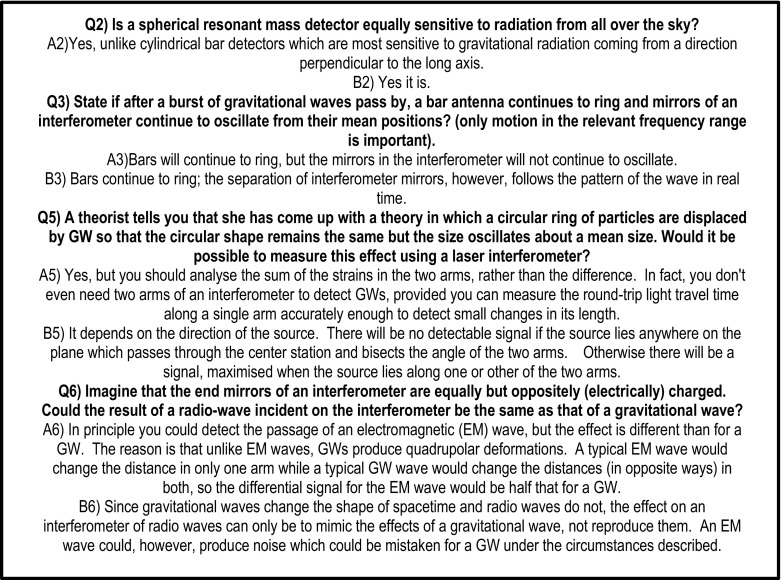
Imitation game with GW physics as the target expertise

Collins, who claimed he had interactional expertise in GW physics took such a test. A contributory expert in GW physics asked him seven technical questions about GW physics via email with a ‘postman’ as the conduit for all the mailings so that addresses/identities were hidden. The same questions went to another contributory expert in GW physics. Mathematical questions were banned. Both participants returned their answers and the completed dialogues were then sent to nine other contributory experts in GW physics to judge who was who—they all knew that one of the participants was Collins. Seven said they could not tell who was who and the remaining two thought that Collins was the contributory expert. Figure 10 shows four of the questions and answers.^7^

We also did experiments on the colour-blind, those with perfect pitch and the blind. These experiments used small numbers of participants rather than a single individual. We wanted to test the theory of interactional expertise assuming that those who spent most time immersed in the linguistic discourse of the complementary group would be better at passing as members of that group. Thus, we compared the ability of the blind to pass as sighted with the ability of the sighted to pass as blind, the blind spending all their lives talking to the sighted whereas the sighted don’t spend much time talking to the blind. As hypothesised, the blind did better at passing as sighted than the sighted at passing as blind, the difference being striking—86 %:13 %. Readers will be able to work out who succeeded best when it comes to the colour-blind versus the colour-perceiving and the ‘pitch-blind’ versus pitch perceiving.

The Imitation Game (we use capitalisation to indicate the research tool), has now been developed so it can be played on a large scale combining it with the idea of the social survey. This is certainly an activity distant from philosophy. Thus we play 200 or so games in various countries to indicate such things as the level of understanding of minorities by mainstream populations taking high pass rates in IGs to indicate good understandings. The topics are such as religion and sexuality. We have also found that in South Africa whites understand blacks much better than blacks understand whites and are working on why this is the case since the opposite was argued to be the case in the US. It may be something to do with changing power relations.

## Interactional Expertise and Embodiment

The experiments on the blind, colour-blind and so forth support the theory of interactional expertise and go a little way to supporting what we call the ‘strong interactional hypothesis—a ‘bold conjecture’, as Popper would call it—which claims that ‘a person with maximal interactional expertise would be indistinguishable from a person with contributory expertise in any test involving linguistic discourse alone’. We provide as an example of the implications of the bold conjecture:

Imagine a person who has been blind and confined to a wheelchair from birth. The claim is that such a person could acquire a practical understanding of tennis solely from extended and intensive discussion of tennis in the company of tennis players without watching tennis or stirring from their wheelchair; such a person could, in principle, understand tennis as well as someone who had played it all their lives.

A bold conjecture does not have to be true to guide further thinking and research. Thus this example has led to it being pointed out that certain congenital limitations affect the early development of the brain—feral children can never learn to speak fluently because the relevant part of their brain has not been developed in infancy—and so there might be aspects of tennis that could not be understood by someone who had certain limitations from birth not because they had no exposure to tennis but because their brain may not have had the opportunity to develop fully. This intimates that claims about interactional expertise should be limited to those who have not or cannot engage in certain practices from early in life rather than from birth.

These experiments and thoughts lead one to notice a crucial distinction that seems absent from the philosophical literature that deals with the importance of the body to the development of conceptual life. Our Wittgensteinian/Winchian starting point, of course, leads us to understand the development of forms-of-life as resting on the intimate relationship of concepts/language and practices whereas the ‘separation principle’ pulls them apart. How is this tension reconciled? The answer is that at the collective level language and practice are inextricable but they can be pulled apart for individuals. There could be no tennis language for the wheelchair bound to learn if members of the tennis collectivity did not play tennis but the individual in the wheelchair can still learn to understand tennis from the spoken discourse alone. There could be no GW physics without all the specialists practising their specialties and feeding their understandings into the discourse but the spoken discourse can still be acquired by an individual without that individual practising anything. To whatever extent the embodiment thesis explains how we think collectively it does not explain the individual acquisition of collective understandings any more than the way the English language has developed explains how I as an individual learned English. And this is vital or we would not be able to understand how those with serious physical limitations are able to speak their native language fluently (or, as has been mentioned earlier, how human societies work).

## Appendix: SEE Publications

Here the description of publications follows the order of the paper and some may appear more than once. I apologise for the Collins-centricity but, though a number of authors are involved, the field is young enough for me to have had a hand in most of the founding papers. A search for the term ‘interactional expertise’ on Google will reveal a flourishing secondary literature and citations to Collins and Evans (2002) and (2007) together are approaching 3000.

SEE is first mentioned in Collins and Evans (2002), but that paper is addressed to the science and technology studies community; we attempt to persuade them to turn their attention to the expertise of scientists so as to recapture a sense of their special place in the social and cognitive world. The ‘fractal model’ is set out in Collins (2011) though it draws on Collins and Kusch (1998). For a large book on the sociology and history of gravitational wave detection see Collins (2004a).

The idea that expertise has three dimensions and the expertise space diagram can be found in Collins (2013). There I also try to reconcile sociology, philosophy and psychology.

The Periodic Table of Expertises is explained at great length in Collins and Evans (2007). A set of papers that covers many aspects of this kind of expertise analysis can be found in Collins (ed) (2007)—a special issue of *Studies in History and Philosophy of Science*. That special issue contains papers on expertise in management (Collins and Sanders 2007) and on how the notion of interactional expertise can be applied to interdisciplinarity (Collins et al. 2007). It also contains a paper on why removing mathematics from the GW imitation game was not a problem since mathematics is not central to every physicist though it is central to physics as a whole (Collins 2007)—another illustration of the difference between collectivities and individuals.

Interactional expertise is first mentioned in Collins and Evans (2002) but the idea is first worked out in depth in Collins (2004b). That paper puts forward the idea that the philosophical tension between language and practice is there only because language has been treated as a set of formal propositions rather than a practice in itself. The paper draws on an earlier source discussing the idea of interactional expertise before the term was invented, namely Collins (1996), where it is introduced in the course of a review of Hubert Dreyfus’s book, *What Computers Still Can’t Do.* The concept is used there, and in some subsequent articles, in a critique of Dreyfus’s view that computers cannot be intelligent unless they have bodies. For a paper that draws together the multiple streams that feed into the idea of interactional expertise and some of the places it is going, see Collins and Evans (2015). For a paper on contributory expertise in the context of technological decision-making in the public domain see Collins et al. (2016).

For an introduction to Imitation Games see Collins and Evans (2014) and for a deep discussion of the large scale game see Collins et al. forthcoming.

For interactional expertise and embodiment see also Collins’s (2000), contribution of Dreyfus’s festschrift and Selinger et al. (2007), which includes a contribution from Dreyfus himself.

## Footnotes

1 The sources are Winch (1958) and Wittgenstein (1953).

2 See Bloor (1976/1991, 1983) and Collins and Kusch (1998).

3 Rather than putting SEE references throughout the text I will locate them in a short "Bibliographical Appendix" at the end which can be used by those who want to explore the topic further.

4 The psychologist Chi (2006) says that individuals go through six stages of increasing sophistication as they become expert while the philosophers, Stuart and Hubert Dreyfus (1986), have a very influential ‘five stage model’ of the development of expertise.

5 Goldman (2001), deals with similar problems though our reading of his work is that he is more concerned with what we call ‘meta-criteria’ than with more general meta-expertise. For an alternative treatment see Collins (2014).

6 That chimps and dolphins might possess vestigial languages makes no difference to the argument.

7 The event and result was published as a news story in *Nature* (Giles 2006). An updated version of the test is reported in Chapter 14 of Collins (2017).
